# Telemedicine Services for the Arctic: A Systematic Review

**DOI:** 10.2196/medinform.6323

**Published:** 2017-06-28

**Authors:** Ashenafi Zebene Woldaregay, Ståle Walderhaug, Gunnar Hartvigsen

**Affiliations:** ^1^ Department of Computer Science University of Tromsø - The Arctic University of Norway Tromsø Norway; ^2^ SINTEF Digital Software Engineering, Safety and Security Tromsø Norway; ^3^ Norwegian Centre for e-Health Research, University Hospital of North Norway Tromsø Norway

**Keywords:** telemedicine, telehealth, health services accessibility, extreme cold, arctic regions, accidents

## Abstract

**Background:**

Telemedicine services have been successfully used in areas where there are adequate infrastructures such as reliable power and communication lines. However, despite the increasing number of merchants and seafarers, maritime and Arctic telemedicine have had limited success. This might be linked with various factors such as lack of good infrastructure, lack of trained onboard personnel, lack of Arctic-enhanced telemedicine equipment, extreme weather conditions, remoteness, and other geographical challenges.

**Objective:**

The purpose of this review was to assess and analyze the current status of telemedicine services in the context of maritime conditions, extreme weather (ie, Arctic weather), and remote accidents and emergencies. Moreover, the paper aimed to identify successfully implemented telemedicine services in the Arctic region and in maritime settings and remote emergency situations and present state of the art systems for these areas. Finally, we identified the status quo of telemedicine services in the context of search and rescue (SAR) scenarios in these extreme conditions.

**Methods:**

A rigorous literature search was conducted between September 7 and October 28, 2015, through various online databases. Peer reviewed journals and articles were considered. Relevant articles were first identified by reviewing the title, keywords, and abstract for a preliminary filter with our selection criteria, and then we reviewed full-text articles that seemed relevant. Information from the selected literature was extracted based on some predefined categories, which were defined based on previous research and further elaborated upon via iterative brainstorming.

**Results:**

The initial hits were vetted using the title, abstract, and keywords, and we retrieved a total of 471 papers. After removing duplicates from the list, 422 records remained. Then, we did an independent assessment of the articles and screening based on the inclusion and exclusion criteria, which eliminated another 219 papers, leaving 203 relevant papers. After a full-text assessment, 36 articles were left, which were critically analyzed. The inter-rater agreement was measured using Cohen Kappa test, and disagreements were resolved through discussion.

**Conclusions:**

Despite the increasing number of fishermen and other seafarers, Arctic and maritime working conditions are mainly characterized by an absence of access to health care facilities. The condition is further aggravated for fishermen and seafarers who are working in the Arctic regions. In spite of the existing barriers and challenges, some telemedicine services have recently been successfully delivered in these areas. These services include teleconsultation (9/37, 24%), teleradiology (8/37, 22%), teledermatology and tele-education (3/37, 8%), telemonitoring and telecardiology (telesonography) (1/37, 3%), and others (10/37, 27%). However, the use of telemedicine in relation to search and rescue (SAR) services is not yet fully exploited. Therefore, we foresee that these implemented and evaluated telemedicine services will serve as underlying models for the successful implementation of future search and rescue (SAR) services.

## Introduction

The advent of information and communication technology (ICT) has revolutionized various aspects of health care services, including the practice of telemedicine. Nowadays, telemedicine services have a great impact on making a patient’s life easier by allowing remote diagnosis and treatment without the constraints of distance and time. Many groups have performed and evaluated different clinical trials onshore and reported the success of telemedicine services [1-5]. However, these success stories are limited to onshore situations, where there are adequate infrastructures. In contrast, maritime telemedicine has received little attention despite the increasing number of seafarers from various nations around the world [6,7]. For example, Duffy’s review [8] regarding the offshore dental problem of Shell Expro indicates that most of the dental problems that resulted in evacuation were preventable while on-board. This study clearly shows that a considerable number of offshore workers suffer from untreated dental diseases, resulting in unnecessary evacuations with associated costs [8]. Moreover, Guitton [7] has assessed the availability of online resources associated with maritime health. The findings show that there is a lack of such resources, and when they exist, they suffer from poor content in terms of understandability and actionability by general audiences, when compared with other medical scenarios. Adoption of onshore technology and research results in offshore conditions might seem like a quick remedy for the case. However, this requires careful investigation of each service so as to identify the underlying difference between the onshore and offshore scenarios. According to Guitton [9], maritime and onshore telemedicine can be convergent and divergent with respect to differences in structure, practices, and policy. Structural differences mainly include differences in data transfer capability (eg, onshore: easy due to availability of enough bandwidth; offshore: difficult due to absence of enough bandwidth), levels of literacy (eg, onshore: mostly between health professionals; offshore: between laymen (seafarers) or paramedics and professionals), supplementary medical assistance (eg, onshore: limited time delays before assistance arrives; offshore: very long time delays before assistance arrives). Practical differences include health records (eg, onshore: complete health records; offshore: incomplete health records), language (eg, onshore: mostly patient’s mother tongue; offshore: potentially not and typically multilingual because of crossing of multiple boundaries), legal context (eg, onshore: typically deployed within a single nation; offshore: multiple nations may be involved), and complementary medical exams (eg, onshore: many; offshore: almost impossible). Policy differences mainly include policy dynamics (eg, onshore: relatively new and fast growing; offshore: old and stable), and research target (eg, onshore: targets of major research, offshore: very few) [9]. Therefore, it is necessary to identify these differences and carefully review them before transferring technology, research results, and experiences to offshore conditions [6,9]. Moreover, Pedersen et al [5] highlighted the different factors, namely, access, quality of care, cost effectiveness, and emergency care, for transferring knowledge from onshore to offshore, by justifying the case with the medical application tested and implemented on an operative basis in Northern Norway. Despite these differences, it is important to note that there is an area where direct adoption of onshore telemedicine services can be fruitful, such as patient-targeted telemedicine interventions, radio-consultation equipped with pictures and video, and video conferencing [9]. Furthermore, research results and experiences from within a similar environment, such as extreme weather conditions like those in Antarctica, can be one area to investigate when developing telemedicine solutions for the Arctic [4].

Despite the increasing number of fishermen and other seafarers, maritime working conditions are characterized by an absence of access to health care facilities [6,7]. There are factors that affect successful implementation of maritime or offshore telemedicine in the Arctic, including long distance, extreme weather conditions, absence of good communication coverage, and the time required for search and rescue (SAR) helicopters to reach the Artic, which reduce the possibility of medical evacuation (MEDEVAC) [6,10-12]. According to AH Gundersen, Senior adviser at The Joint Rescue Coordination Centre of Northern Norway (JRCC NN) in Bodø, time is considered to be the scarcest resource in an emergency situation, particularly in the Arctic, because of the long distance and harsh environmental conditions [13]. Moreover, the absence of on-board trained nurses or physicians, limited equipment and medicine, and onshore professional advice limited to only radio medical advice, further aggravates the situation [6]. Nowadays, there is a growing interest in the Arctic region in connection with the discovery of huge gas and oil resources [14,15]. However, it remains to be solved how appropriate health care services can be offered in this area. Remoteness, Arctic winter darkness, extreme weather conditions, and poor communication network coverage in the area pose a challenge for the use of remote telemedicine services [4,10,15-18]. Moreover, during a large-scale accident such as a shipwreck, the temperature of the region, which might go below −40^o^ C [16,19,20], further reduces the chances of victims surviving until search and rescue teams arrive. This situation has posed additional challenges for the implementation of successful emergency telemedicine services in the Arctic region.

Generally speaking, a review of telemedicine services in the context of maritime conditions, remote and extreme weather setting are inadequate. However, there are several reviews on telemedicine services regarding onshore remote accident and emergency services that have been published [21-24]. For example, Keane [22] conducted a comprehensive review on the success of telemedicine in the scope of accident and emergency. Amadi et al [23] also conducted a review to examine the history and existing applications of telemedicine in pre-hospital environments, where telemedicine is believed to extend the reach of specialist services to handle pre-hospital care of acute emergencies, in cases where treatment delays may affect the clinical outcomes. The purpose of this review is to assess and analyze the status of telemedicine services, focusing on services that can fit the diverse nature of the Arctic region (environment), which is offshore, remote, and has extreme weather conditions. Moreover, it presents state of the art systems of implemented telemedicine services in remote Arctic regions and also analyses these services within the context of search and rescue services (SAR).

## Methods

For the purpose of the study, we conducted a rigorous literature search between September 7 and October 28, 2015, through various online databases. The searched databases included Google Scholar, PubMed (Medline), Science Direct, ACM Digital Library, IEEE Xplore, Onepetro, the Journal of American Medical Informatics Association (JAMIA), the Journal of International Maritime Health and the Journal of Telemedicine and Telecare. Furthermore, additional articles are also extracted from the reference lists of the selected papers in order to get a complete overview of the state of the art systems. Peer-reviewed journals and articles published between 1995 and 2015 were considered. The inclusion and exclusion criteria were setup through rigorous discussion and brainstorming among the authors. Several combinations of the terms “Arctic,” “oil and gas,” “shipping,” “telemedicine,” “search and rescue,” “maritime medicine,” “offshore,” “extreme weather,” and “telehealth” were used during the search. The search strings were combined using “AND” and “OR” for a better searching strategy. The titles, keywords and abstracts were used for a preliminary filter with our selection criteria to identify relevant articles, we then reviewed full texts for articles that seemed relevant. Some predefined categories were used for information extraction from the selected literatures, which were defined based on previous research and also further elaborated upon via iterative brainstorming. Please note that the words “telemedicine” and “telehealth” are used interchangeably throughout our discussion.

### Inclusion and Exclusion Criteria

To be included in the review, the studies had to have a direct involvement of telemedicine and eHealth in the following scenarios: maritime or offshore conditions and shipping, oil and gas, search and rescue, Arctic and extreme weather conditions, and remote accidents and emergencies. The studies were expected to describe solutions and implement and evaluate telemedicine services to be included in the review. Therefore, studies that were outside of the scope mentioned above were excluded from the review. Moreover, studies in all other language but English are excluded from the review.

### Data Categorization and Data Collection

Information was extracted from the literature based on defined categories (variables). These categories were defined based on previous research, literature reviews, and also further elaborated upon via iterative brainstorming. The categories included were solely defined to assess, analyze, and evaluate the current status of telemedicine services in maritime or offshore, remote accident and emergency, and extreme weather (Arctic, Antarctica) settings. The categories we decided to include were as follows:

- Type of Circumstance: This category defines the circumstances in which the telemedicine services were delivered during the study period, that is, maritime or offshore, remote accident and emergency, or extreme weather (Arctic, Antarctica) conditions.
- Communication Link: This category defines the communication link used to facilitate the practice of telemedicine in these circumstances. This includes various communication networks such as satellite, radio, mobile, dial-up, DSL, and broadband.
- Telemedicine Modalities: This category defines the underlying modalities that enable telemedicine services to be provided. It includes different means of providing telemedicine services such as audio (ie, radio and telephone), video (ie, videoconferencing), text (ie, email), and picture (ie, still images).
- Telemedicine Services: This category defines the type of telemedicine services delivered during the study period.

### Literature Evaluation

Studies were included and evaluated if and only if they presented solutions for the implementation and evaluation of telemedicine services within these scenarios. Evaluation and analysis of the included literature were conducted based on the above defined categories. The first analysis was conducted, based on the first category, to evaluate the type of circumstances (ie, maritime and offshore, extreme weather (Arctic region and Antarctica), and accident and emergency) in which the telemedicine services were delivered during the study period. A trend comparison of published literature with five years intervals was conducted to assess and compare the trends of these circumstances against the publication years. The percentages were computed based on the number of counts (n) of each type of circumstance against the publication years. For example, let us take the interval 1995-2000; the percentage was calculated based on the count of these circumstances (maritime, offshore, and remote; Arctic and extreme weather conditions; accident and emergency) addressed by the literature within this interval. The second analysis was conducted, based on the second category, to evaluate and compare the successful communication means used in these types of circumstances. The percentages were calculated based on the number of counts (n) of each subcategory of communication means used in all these types of circumstances. The third analysis was conducted, based on the third category, to evaluate the telemedicine modalities used in these types of circumstances. The percentages were computed based on the number of counts (n) of each subcategory of telemedicine modalities used in all these types of circumstances. The fourth analysis was conducted, based on the fourth category, to evaluate the type of telemedicine services delivered in the included literature in these types of circumstances. The percentages were calculated based on the number of counts (n) of each telemedicine service addressed in each type of circumstance. We noted the possibility that an article could address multiple circumstances, multiple types of communication links and modalities, and provide multiple services. Therefore, the number of characteristics reported in the Multimedia Appendix 1,Tables 1-5, and Figure 2 could exceed the number of articles reviewed.

**Figure 1:**
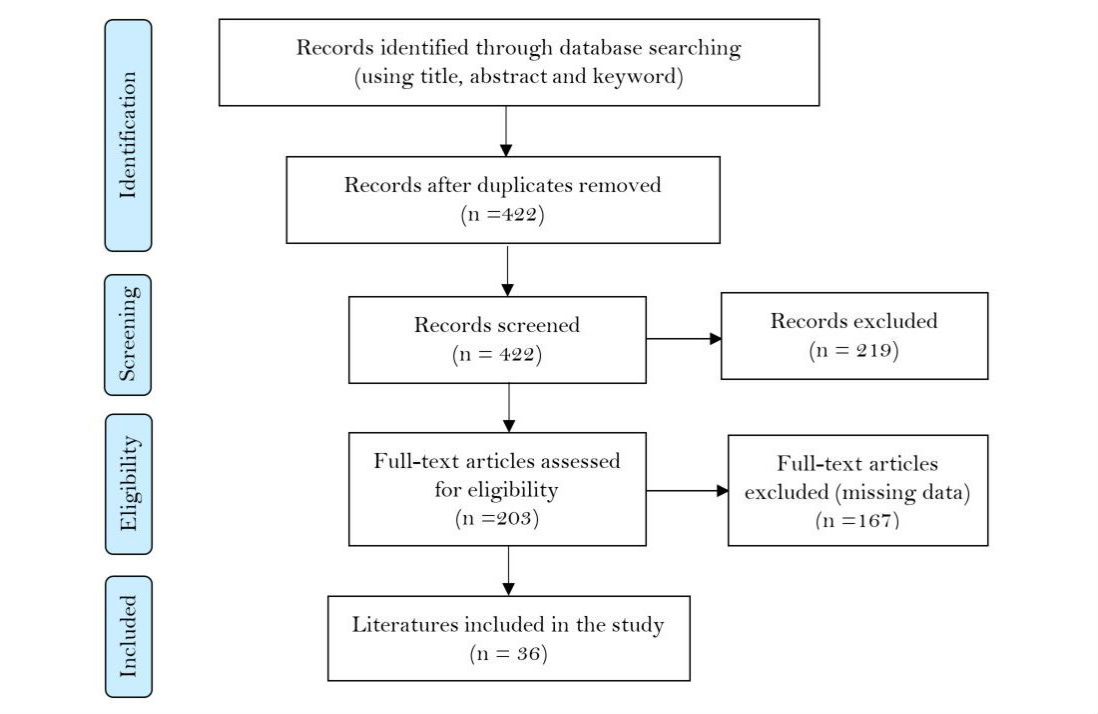
Flowchart of the review process.

## Results

### Relevant Literatures

We vetted the first hit using the title, abstract, and keywords and retrieved a total of 471 papers. After removing duplicates from the group, 422 records remained. Then the authors did independent assessments of the articles, screening based on the inclusion and exclusion criteria, as illustrated in Figure 1, which eliminated another 219 papers, leaving 203 relevant papers. After a full text assessment, 36 articles were left, which were critically analyzed (as shown in Multimedia Appendix 1). The inter-rater agreement was measured using Cohen Kappa test, and disagreements were resolved through discussion. The “missing data” in Figure 1 depicts that the study either does not have a solution for the implementation, design, and evaluation of the system or is not within the context of the review.

**Figure 2:**
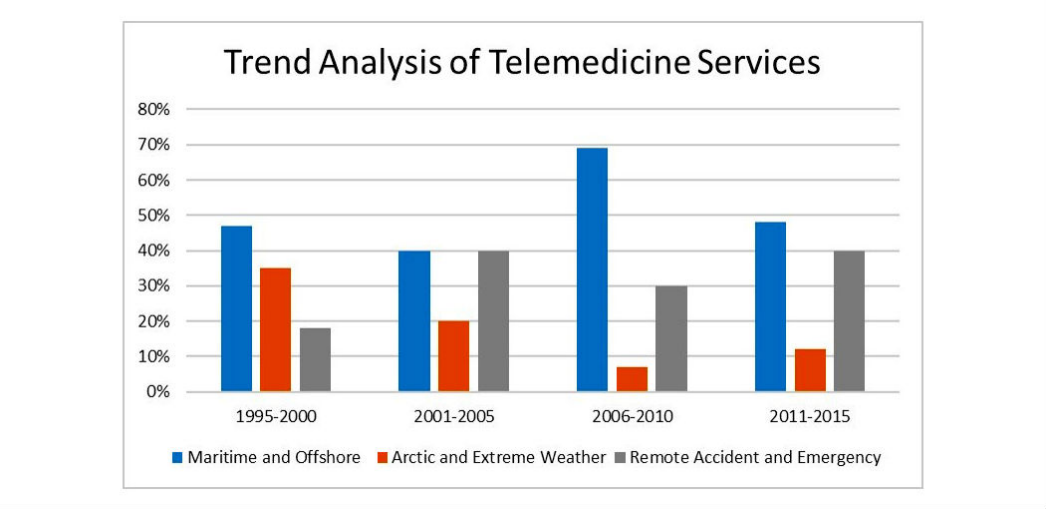
Comparison of published literature in telemedicine services within the context of maritime and offshore, Arctic and extreme weather, and remote accident and emergency during the last 20 years.

### Evaluation and Analysis of the Literature

The analysis and evaluation of the included articles are given in Figure 2 and Tables 1-5. The evaluation and analysis presented in this section are entirely based on the data categorizations given above.

The research effort put forward in the development of telemedicine services during the past 20 years in these different circumstances are shown in Figure 2. As shown in Figure 2, telemedicine has seen an almost steady progress in maritime and offshore services, peaking between 2006 and 2010. Moreover, telemedicine has also seen the same trend in remote accidents and emergencies as in maritime and offshore settings. However, telemedicine services in Arctic and extreme weather conditions have seen large fluctuations depicting the challenges imposed by bad weather, absence of communication networks, and absence of Arctic-enhanced telemedicine equipment in this region.

Various means of communication (networks) can be used for the implementation of telemedicine services. These include satellite, mobile (GSM, GPRS, and CDMA), ISDN, broadband, and Virtual Private Network (VPN), depending on availability and suitability. As shown in Table 1, satellite is the most used means of communication (20/70, 28%). The second most used means of communication is mobile (19/70, 27%), which includes GSM, GPRS, CDMA, and so on. Radio, VPN, and others are ranked third (13/70, 19%). ISDN is ranked fourth (11/70, 16%), followed by broadband (7/70, 10%).

**Table 1:** Comparison of communication links used in the published literature from 1995 to 2015.

Communication means	Usage count	Usage percentage
Satellite	20	28%
Mobile (GSM^a^, GPRS^b^, CDMA^c^)	19	27%
Others (Radio, VPN^d^, and others)	13	19%
ISDN^e^	11	16%
Broadband	7	10%

^a^GSM: Global System for Mobile Communication.

^b^GPRS: General Packet Radio Service.

^c^CDMA: code division multiple access.

^d^VPN: Virtual Private Network.

^e^ISDN: Integrated Services Digital Network.

Telemedicine services can be developed based on a different approach known as telemedicine modalities. This includes the use of video, still images, bio-signal or medical data, audio, and e-mail. As shown in Table 2, video is the most frequently used (25/93, 27%) form of delivering telemedicine. Transmission of still images is the second most used (23/93, 25%) form of delivering telemedicine. Audio (including radio and telephone) ranked third (18/93, 19%), followed by text (including email) (16/93, 17%), and others (Bio-signal transmission including ECG waveform, medical data) (11/93, 12%) ranked last.

**Table 2:** Comparison of published papers based on modalities of telemedicine from 1995 to 2015.

Type of modality	Usage count	Usage percentage
Video (VTC and others)	25	27%
Still pictures	23	25%
Audio (Radio, telephone, and others )	18	19%
Text (Email and others)	16	17%
Others ( Bio-signal, medical data, and others)	11	12%

There are different types of onshore telemedicine services tested and evaluated in emergency and non-emergency contexts, including teleradiology, teleconsultation, teledermatology, telecardiology, telemonitoring, tele-education, tele-ENT, and others. The extent and types of such services used in the context of maritime and offshore, accident and emergency, and extreme weather (Arctic region and Antarctica) are evaluated and compared as shown in Tables 3-5.

Among the telemedicine services that are developed in maritime and offshore, teleconsultation is the most used (9/32, 28%); see Table 3. This includes both real-time (online) and offline teleconsultation that are considered and used in the studies. The second most used type of telemedicine service is Telecardiology (telesonography) (8/32, 25%). Others (7/32, 22%) takes the third rank, which consists of data sharing and decision-making, telepointing, telepresence, and others. Teledermatology (3/32, 10%) ranked fourth, followed by tele-ENT (2/32, 6%), radio-medical advice (2/32, 6%), and tele-education (1/32, 3%).

**Table 3:** Comparison of telemedicine services in the maritime and offshore context reported in the literature from 1995 to 2015.

Type of service	Usage count	Usage percentage
Teleconsultation	9	28%
Telecardiology (Telesonography)	8	25%
Others (Telepointing, Telepresence, Data sharing, and Decision-Making)	7	22%
Teledermatology	3	10%
Tele-ENT	2	6%
Radio medical advice	2	6%
Tele-education	1	3%

Regarding the telemedicine services in extreme weather (Arctic region and Antarctica), as shown in the Table 4, other forms of telemedicine services such as tele-interpretation, tele-ambulance, data sharing and decision-making and others are the most used forms of telemedicine (10/37, 27%). Teleconsultation is the second most used form of telemedicine (9/37, 24%) followed by teleradiology (8/37, 22%). Both teledermatology and tele-education ranked fourth most used (3/37, 8%). Tele-ENT is the fifth most used service (2/36, 6%). Telemonitoring and Telecardiology (telesonography) were ranked equally as the sixth most used telemedicine services (1/37, 3%).

**Table 4:** Comparison of telemedicine services in the extreme weather context reported in the literature from 1995 to 2015.

Type of service	Usage count	Usage percentage
Others (Tele-interpretation, Tele-ambulance, Data sharing and Decision-Making, and others)	10	27%
Teleconsultation	9	24%
Teleradiology	8	22%
Tele-education	3	8%
Teledermatology	3	8%
Tele-ENT	2	5%
Telemonitoring	1	3%
Telecardiology (Telesonography)	1	3%

As shown in Table 5, among the telemedicine services implemented within the context of accident and emergency, Others, which includes telepresence, tele-ophthalmology, Tele-EMS, tele-ambulance, and others are the most used means of delivering telemedicine during accident and emergency (19/60, 32%). Teleconsultation is ranked the second most used means of delivering telemedicine during accident and emergency (16/60, 27%). The third most used is telecardiology (telesonography) (7/60, 12%), followed by teleradiology (5/60, 8%), teledermatology (4/60, 7%), and telemonitoring (3/60, 5%). The least used are radio medical advice, tele-education, and Tele-ENT, each of which accounts for 3% (2/60).

**Table 5:** Comparison of telemedicine services in the remote accident and emergency context reported in the literature from 1995 to 2015.

Type of service	Usage count	Usage percentage
Others (Telepresence, Tele-ophthalmology, Tele-EMS, Tele-ambulance, and others)	19	32%
Teleconsultation	16	27%
Telecardiology (Telesonography)	7	12%
Teleradiology	5	8%
Teledermatology	4	7%
Telemonitoring	3	5%
Tele-ENT	2	3%
Tele-education	2	3%
Radio Medical Advice	2	3%

## Discussion

### Principal Findings

Telemedicine has a vital role in delivering health care services without the constraint of time and space. According to Ekeland et al [25], telemedicine has been shown to be cost effective and also to have a positive impact in various scenarios such as therapeutic effects, health care services efficiencies, and technical usability. It is an indisputable fact that telemedicine also has a transformative power on health care delivery in extreme weather, maritime/offshore, and remote emergency/accident scenarios [26]. Despite the increasing number of fishermen and other seafarers, maritime working conditions are mainly characterized by an absence of access to health care facilities. The condition is further aggravated for fishermen and seafarers who are working in the Arctic regions. Even if onshore telemedicine has been a success, its success offshore is limited. This is because of various reasons such as the absence of good communication networks, the absence of trained paramedics, bad weather conditions, and others. However, irrespective of these limitations, maritime and emergency telemedicine services have recently been successfully delivered in the Arctic, Antarctica, and other areas with extreme weather conditions. These services include teleconsultation, teleradiology, teledermatology and tele-education, telemonitoring, telecardiology (telesonography) and others (including tele-interpretation, tele-ambulance, data sharing and decision-making, and others). These services used various means of communication networks such as satellite, mobile, and radio along with different modalities such as video, still images, audio, and medical data.

### Telemedicine Services in Maritime and Offshore Conditions

This section presents the status of telemedicine in maritime and offshore conditions and also identifies successful deployment of telemedicine services within these circumstances. The working conditions that exist in maritime settings are characterized by absence of access to health care facilities due to various reasons. Horneland [6] describes the limitations that hinder access to such facilities, which include the distance and time that SAR helicopters need to reach the scene, which reduces the possibility of MEDEVAC. To remedy these challenges, telemedicine is the sole choice for delivering health care services at the scene within a short response time. For instance, telemedicine services have been in use at different offshore petroleum installations and also in the Norwegian maritime fleet since 2006. Telemedicine has a great advantage for offshore personnel by providing them with access to better health care within a short response time [27]. In emergency situations, physicians onshore can thoroughly examine a patient, allowing them to accurately assess the patient’s condition and develop a plan for care [28]. Moreover, telemedicine can provide advantages for companies by minimizing unnecessary medical evacuation and ship diverting for seeking medical assistance [28]. For example, Patel and Stoloff et al [29,30] conducted a cost benefit analysis of shipboard telemedicine and reported the benefit of telemedicine for a ship found at a distance of over 200 nautical miles (370 km) from shore; in such cases, the use of helicopter is found to be too costly. However, the absence of on-board trained nurses or physicians, limited equipment and medicines, availability of limited bandwidth, lack of trained paramedics, lack of complete health records, language barriers, lack of complementary medical exams, and the limitation of professional advice to only radio medical advice remains to be a challenge for the implementation of successful telemedicine services [6]. Horneland [6] clearly indicates the area that should be taken into account for the improvement of telemedicine within the context of maritime health care. Accordingly, education and training of seafarers takes priority for improvement of the services [6,31]. Additionally, preparing medical handbooks or manuals for seafarers, which are like supplements to the texts on the market, can enhance the quality of health care delivery through telemedicine. Pre-sea and periodic medical examinations are considered necessary to reduce the high risk of medical emergencies while on board [6,28]. Anscombe [28] also emphasized the need for having a proper health fitness standard that should be met before joining the task force. Recently, several research groups have performed and evaluated different clinical trials on an onshore basis and reported the success of telemedicine services [1-5]. However, this success is limited to onshore, where there is good infrastructure. By contrast, maritime telemedicine has received little attention despite the increasing number of seafarers from various nations around the world [6]. Therefore, it is deemed necessary to consider a space for adapting research results, technologies, and experiences from onshore to offshore scenarios. For example, Horneland [6] highlights the necessity of having a careful review before adapting onshore telemedicine services to offshore scenarios by justifying the case with the use of Electrocardiography (ECG) and thrombolysis. Guitton [9] gives a brief explanation about the convergence and divergence points of maritime and onshore telemedicine services by justifying the three major differences, namely structural, practical, and policy differences. Furthermore, it highlights concepts and issues for identifying these differences for better transfer of technology and research results from onshore to offshore settings. However, in spite of these differences, there are areas where direct adoption of onshore telemedicine services can be fruitful, such as patient-targeted telemedicine interventions, radio-consultation equipped with pictures and video, and video conferencing [9].

Despite the little attention maritime telemedicine has received over the past years, recently, some researchers and companies have performed studies on adopting and improving maritime telemedicine. For instance, Aujla et al [32] conducted a study on a rationalizing effort of ship to shore radio medical advice for the UK. According to Aujla et al [32], a radio medical advice is most effective when the demographic data of the population at risk are identified. Auditing the nature and frequency of medical emergencies on various types of vessels should create a basis for future recommendation regarding the minimum medical facilities needed. Furthermore, a guide for handling various conditions, that is, treatment and alternative strategies for handling the inability to evacuate patients because of bad weather should be developed. This includes provision of adequate training for the medical staff providing the services at both sites. Likewise, Saipem’s Medical Department also conducted a project to investigate and develop telecardiology so as to provide remote sites with practical support in cardiology and to extend the company’s preventive approach toward cardio-vascular diseases [33]. The study has shown that telecardiology could, in fact, prevent a lot of unnecessary medical evacuations. This includes online assessment of suspected acute conditions, early detection of heart problems, and adequate filtering and priority grading of referrals for patients requiring further investigation, while reducing the load of unnecessary referrals for primary diagnosis [33]. Moreover, MERMAID is another breakthrough telemedicine project, which is a telematics-based response to the EU requirement for “long distance medical consultation” to safeguard the health and safety of maritime workers and isolated populations. The developed system is capable of delivering an integrated 24-hour multilingual worldwide emergency service to transfer medical expertise via satellite and ground-based ISDN networks [26,34-36]. The connectivity of the system is realized by combining various communication links, such as mobile satellite technologies, VSAT technologies, and ISDN protocols. The project has explored almost every category of telemedical application (audio and video conferencing, multimedia communications, flat file and image transfer with low-, medium-, and high-bandwidth data requirements), along with a full range of network choices (digital land lines, cellular or wireless, satellite, and broadband) and analysis in terms of the cost or performance trade-offs inherent to them. Moreover, it provides a variety of services, among which the notable one is electronic transmission of medical information via ISDN-based video conferencing. In addition, medical telecommunication software is considered that includes a medical record system that can guide the user through patient history and support objective examination coupled with a multimedia HELP function capability, that is, text and illustrations, based on WHO and the EU (DG V) requirements for help at sea, to guide paramedics through all the operations with a teleconsultant. Anogianakis et al [34] conducted a study that provides a means for the training and education of seafarers through the use of the MERMAID medical communications system as this is the firmest basis for the promotion of the proper practice of telemedicine at sea.

Providing access to patients’ health care records can improve the health care process, thereby improving the decision-making ability of caregivers without the constraint of distance and time, whether it is at the bedside or at remote locations. One of the challenges within maritime telemedicine is the lack of complete shared health records. In this regard, Thorvik et al [12] developed and tested a telemedicine prototype known as a virtual examination room, which is an example of software for sharing medical data, enabling collaboration in different situations and based on optimal workflows between the offshore and onshore medical facilities. The concept of the virtual examination room is to give freedom and interconnectedness among the medical experts, the hospital, and the offshore nurse to simultaneously see, interpret and discuss the medical information available in the virtual examination room that has been retrieved from the connected medical devices. Likewise, Anogeianaki et al [37] also implemented a minimum medical emergency data-set (MMEDS), which enables patients to record their own health status so that information can be available for any treating physicians, irrespective of where the patients are located. The system was tested and evaluated across the Greek-Bulgarian border. Besides, Boultinghouse et al [38] reported the use of electronic medical records (EHR) during health care services delivery for oil and rig workers and implemented an electronic medical record that can keep all the health information safe, organized, and accessible over any distance, eliminating the problems and delays of a paper-based record system. This kind of shared electronic health record was provided for medics in offshore settings. Moreover, Amenta et al [31] developed an electronic medical file for each patient assisted, where the data would be updated following every radio contact with a ship or a plane in flight. Furthermore, Anogianakis et al [35] also developed software that can provide a medical record system, which can guide the user through patient history and objective examination. In addition, it provides a database that contains all the information on the vessel’s stocks of medicine and medical equipment.

Assessment of user satisfaction is an important requirement for delivering quality health care services. Therefore, user satisfaction assessment on the use of maritime telemedicine has been conducted. For instance, Dehours et al [39] conducted a study on the CCMM telehealth services, the operators of French Tele-Medical Assistance Service (TMAS). During the study, 385 surveys were e-mailed, of which 165 were completed and used for analyzing user satisfaction. Overall, the result indicates that the satisfaction of on-board caregivers was high; callers were satisfied with the telephone advice, competence of physicians involved, and waiting time for services, oral prescriptions, and medical advice. The study has also given some useful recommendations for successful implementation of on-board ECG and still pictures [39]. Mair et al [40] conducted a telemedicine trial service to analyze the impact of telemedicine on reducing unnecessary evacuation. The system relies on satellite communication to provide a videoconferencing service to diagnose and treat remote oil and rig workers. The study concluded that participating onshore physicians were very satisfied on each occasion with the communications and diagnostic data and image quality, including the ultrasound screening carried out by the rig provider. The study showed that remote specialist advice via videoconferencing should reduce unnecessary and untimely patient evacuation to hospital or onshore for medical check-ups. In addition, Kevlishvili et al [41] studied the effect of teleconsultation on clinical settings. The study used videoconferencing through Skype, email, and still image services to support remote diagnosis and treatment in decision-making. Even though the trial was small, the study concluded that a telemedical solution has a great effect on simplifying remote treatment and diagnosis.

### Telemedicine Services in Extreme Weather Conditions

This section presents the status of telemedicine in extreme weather condition (Arctic, Antarctica, and others) and also discusses the successful deployment of telemedicine services in these circumstances. Working in extreme cold weather has many health complications **.** Extreme atmospheric temperature has major consequences on the body’s thermal reactions and the risk of accidents increases when the ambient temperature falls below 0° C. Despite these health risks, currently there is a great deal of interest in the Arctic region from different companies, professionals, and merchant seafarers in connection with the discovery of huge natural resources. In order to survive in this extreme cold environment, there are services that should be put in place such as good health care services, medical examinations for fitness to work, vaccinations, first aid training for extreme cold conditions, clothing requirements, and provision of other Personal Protective Equipment (PPE) [42]. However, these regions are characterized by an absence of good health care services and weather-enhanced equipment. Due to these limitations, these regions were solely served by air ambulance operation or helicopter evacuation to get medical services from onshore specialists. However, the air ambulance operation has a lot of challenges and drawbacks including winter darkness and foggy weather. For example, Norum et al [18] analyzed air ambulance operations due to cardiovascular disease (CVD) in the Arctic region from 1999 to 2009. The study tried to analyze the challenges faced in the air ambulance operations in the Arctic region, such as long distance, rough weather conditions, and almost no alternatives for landing. According to Norum et al [18], telemedicine for remote consultation and treatment is vital for on-board vessels and rigs. However, various factors hinder the successful development of telemedicine services in the Arctic high north. This is clearly shown by Walderhaug et al [10], with a specific emphasis on the use of telemedicine in the search and rescue operation. According to Walderhaug et al [10], long distance, bad weather conditions, winter darkness, and poor communication infrastructure are some of the challenges highlighted for developing successful telemedicine services.

Despite these facts, there are groups that conducted research on alleviating these challenges. For example, the Baffin Telehealth Project is one of the Canadian High Arctic projects, which is designed to serve the remote communities of the Canadian High Arctic. The project aims at providing better health care access to the communities of the Baffin Region [43] by using different technologies from remote telemedicine systems so as to cope with isolating geography and severe environmental conditions. The system based its development on the use of a high bandwidth satellite communication to offer real-time video conferencing, digital imaging, and various medical diagnostics to support remote health stations on Baffin Island [43]. Moreover, the Mount Logan and Mount McKinley Telemedicine Projects are other examples of telemedicine projects that serve remote environments [43]. Latifi et al [44] also provide a system called the Amazon Virtual Medical Team (AVMT), which uses telemedicine services to provide health care services for swimmers in the Atlantic Ocean. The system relied on advanced technologies and a low bandwidth satellite connection to help an assembled virtual medical team to ensure telepresence 24/7 throughout the mission. Furthermore, Todnem et al [45] presented a project developed by the Statoil company for implementing telemedicine services on all Statoil operated offshore installations on the Norwegian continental shelf (NCS), succeeding in the initial pilot project from 2007-2008. The services provided included videoconferencing for meetings and educational purposes and to spread vital medical information to many locations or installations at the same time, which has been essential during epidemic situations (Swine flu, Noro virus etc). The study also demonstrated that it is possible, using the existing telemedicine equipment, to successfully remotely guide a nurse offshore in focused ultrasound examinations, with the medical doctor or expert located onshore. Similarly, one of the largest oil and gas companies, Shell, has developed remote telemedicine systems for delivering enhanced health care services to its worker [46]. The project proposes remote health care (RHC), which involves an integrated approach for delivery of health care in the Arctic operations. The system meets both the emergency and non-emergency requirements for delivering the best health care services. RHC includes different aspects such as prevention, technology, supplies or equipment, competence, and communication. The RHC serves as a virtual hospital for patients to get treated on board by allowing the physician on board the vessel to communicate real-time with onshore specialists and enabling these onshore specialists to visualize the patient using high-definition mobile cameras. The system is based on an enhanced medical technology for diagnosis and treatment, including the latest in near patient laboratory testing, digital X-Ray and “pocket” ultrasound equipment that is linked to the hospital radiology department via satellite [46]. The study assessed the outcome in Greenland, Siberia, and West Africa.

Regarding telemedicine services in the Antarctic region, various nations have performed trials for providing health care delivery for tourists and groups of researchers. For example, Grant [47] reports the experiences of using telemedicine services in the Antarctic region. According to the report, BASMU in Aberdeen have developed a tool called the Medical Assessment Questionnaire (MAQ), which proves to be effective in communicating with on-board seafarers by minimizing the treatment time, thereby allowing more accurate and error free telediagnosis and possibly reducing medical evacuations [47]. The study has also conducted various telemedicine services including successful transmission of ECG tracings through fax and e-mail for diagnosis, the potential use of thrombolysis, telemetry (even if equipment was shown to be unreliable and had poor battery life), digital x-ray equipment, ultrasound examination, internet-based education and tele-interpretation [47]. According to the report, telespirometry and more useful systems of teleconsultation are believed to be possible **.** Similarly, Ohno and Ohno et al [48,49] also conducted a study for delivering telemedicne solutions to be used by the Syowa Station, Japanese Antarctic Research Expedition (JARE). The developed system was intended to handle various practical cases including emergency cases. This system has shown the success of telemedicine in handling various medical operations such as surgery, orthopedics, ophthalmology, dermatology, internal medicine, urology, and dentistry [48,49]. In addition, Pillon et al [50] also reports the experiences and success of developing a telemedical solution for the principal Italian Antarctic Base at Terra Nova Bay. The system was developed to link the area with the largest Italian hospital, San Camillo in Rome. Full teleconsultation practice via videoconferencing has been developed for consultations with ophthalmic, orthopedic, and radiology specialists. Furthermore, a number of telemedicine projects have been conducted in Alaska [51], including the Alaska Telemedicine Testbed Project (ATTP), Alaska Federal Healthcare Partnership (AFHCP), AFHCP Tele-radiology Project, the Alaska Federal Health Care Access Network (AFHCAN), AFHCAN Telemedicine Hardware and Software, and AFHCAN Connectivity & Network (WAN). Moreover, Hild [51] discusses the challenge and success factor for successful implementation of telemedicine services in Alaska. According to the report, any successful telemedicine services should be developed and designed in consideration with physical infrastructure, training structures, interoperability guidelines, and community interfaces [4]. Hence, Hild [51] also discusses the experiences of telemedicine in Alaska in accordance with these success factors.

### Telemedicine Services in Remote Accident and Emergency Responses

This section presents the status of telemedicine in remote accident and emergency responses along with the search and rescue (SAR) scenarios and also discusses the successful deployment of telemedicine services (status quo) within these circumstances. Telemedicine is an optimal candidate for managing remote accidents and emergencies. However, it should be considered as a support to emergency management and not as a final solution. During an accident or emergency, the various means of communication such as real-time, store-and-forward, and data exchange can be used as either first opinion or second opinion services.

Management of accident and emergency responses are a crucial part of both onshore and offshore health care services. An accident that threatens life and health should get first aid and immediate assistance from the nearby paramedic or specialist. However, sometimes an accident could happen in a remote area such as in the Arctic region, and it takes a significant amount of time to visit a specialist. Any further delay of time in an accident means reducing the survival chances of the victims. Therefore, it is necessary to have a means of treating the victims while in a remote area, at least to prolong the chance of survival. The telemedicine system designed for real-time emergencies has paved the way for a new perspective in remote medical diagnosis [52]. Ensuring safety in the Arctic waters is very challenging because of the remoteness of the region and the lack of contingency planning infrastructure [4]. According to Berg et al [53], so as to reduce the rate of accidents in the Arctic waters, it is necessary to improve regional collaboration, develop additional professional requirements for seafarers, and provide training in order to offer knowledge transfer from seniors working in the region. For example, Buschmann et al [54] described the need for the medical education concept, “SAR-First Responder Sea,” to help paramedics in providing treatment and diagnosis during search and rescue operations. Miller et al [55] also conducted a retrospective review to analyze the potential of emergency nurse practitioners (ENPs) for delivering telemedicine advice for minor injuries. The result is in agreement with Buschmann et al [54], which supports the conclusion that the assessment of all minor injuries through a telemedicine network by medical staff is unnecessary and that an ENP-led service offers a realistic and attractive alternative. Similarly, Boniface et al [56] conducted a study to assess the capability of ultrasound-naive paramedics to obtain interpretable Focused Assessment with Sonography for Trauma (FAST) pictures under the remote guidance of Emergency Physicians (EPs). The result has shown that paramedics with no prior ultrasound experience could obtain FAST images under remote guidance from experienced EPs in less than 5 minutes. This result has a potential advantage for managing remote accidents by treating patients through data transmission [56]. In addition, Bergrath et al [57] investigated the feasibility and effect of pre-hospital teleconsultation to transfer the concept into the emergency medical services. The study was conducted in a real clinical setting by comparing telemedically assisted pre-hospital care (telemedicine group) with the local regular EMS care (control group). The study concluded that teleconsultation is feasible but technical performance and reliability have to be improved. Moreover, the result has shown the future potential of pre-hospital tele-stroke consultation to improve emergency care, especially when no highly trained personnel are on-scene [57]. Furthermore, Brebner et al [58] evaluated a pilot telemedicine network for accident and emergency work. The study assessed the treatment and diagnosis of emergency cases through videoconferencing for a period of 15 months. The study demonstrated that accident and emergency teleconsultations can be technically reliable and effective in reducing the number of patient transfers and in a manner acceptable to the referring clinicians. Similarly, Bowman et al [59] conducted a controlled trial to assess the accuracy of telemedicine in diagnosing and managing eye problems presented to accident and emergency. The study has shown that telemedicine, utilizing video slit lamp images, to be an effective, safe, and accurate method of diagnosing and managing these patients.

Castellano et al [52] conducted a study for handling emergency cases in a pre-hospital environment. The study designed a real-time emergency telemedicine system for remote medical diagnosis (an ambulance) using a hybrid network that enabled secure long-distance communication from an ambulance. Moreover, the study demonstrated a specific scenario by performing an ambulance-based hematological test with regard to an international normalized ratio (INR) using wireless transmission, accurately and in real-time, to the referral hospital. The study reported no significant differences between the ambulance-based and the laboratory-based tests [52].

Kang et al [60] conducted a preliminary study that evaluated the use of a code division multiple access (CDMA)-based emergency telemedicine system to be used by emergency rescuers providing first-aid treatment to patients. The evaluated prototype consisted of equipment for measuring non-invasive arterial blood pressure (NIBP), arterial oxygen saturation (SpO2), six-channel electrocardiogram (ECG), blood glucose concentration, and body temperature. The recorded patient data were transmitted to the doctor’s computer through CDMA and TCP/IP networks using an embedded personal digital assistant (PDA) phone. The result indicates that the systems provided reliable values. Moreover, the feasibility of the prototype was evaluated with 15 real emergency patients on Jeju Island over a two-month period. The measured data were successfully transmitted without significant CDMA connection loss or transmission errors.

Uldal et al [61] developed a mobile telemedicine unit (MTU) for emergency and screening purposes, which included various facilities such as endoscopy, electrocardiography, and digital photography. The mobile telemedicine unit included a modem, a portable PC, an endoscope, an electrocardiogram (ECG) facility, a digital camera, a printer, and an uninterruptible power supply (UPS). In this system, data transmission was implemented using an ordinary telephone line.

Kyriacou et al [62] developed a portable medical device that supported emergency telemedicine by allowing telediagnosis, long distance support, and teleconsultation of mobile health care providers by expert physicians. The system combined both real-time and store and forward facilities by using a telemedicine unit at the patient or emergency site and the expert’s medical consulting at the base unit. This integrated system had a capability to be used in various emergency cases such as being treated in an ambulance vehicle, in a Rural Health Centre and on a navigating ship. The developed system was a “multi-purpose” telemedicine system consisting of two major parts: a telemedicine unit (located near the patient) and a base unit (located at a Central Hospital). The Telemedicine unit mainly consisted of hardware and software components such as a bio-signal acquisition module, image capturing module, main module, and communication module. The system relied on a GSM or GPRS modem and a POTS modem or satellite modem for communication purposes. The system was clinically evaluated and installed and used in two different countries: Greece (ambulance vehicles, rural health centers, ships) and Cyprus (ambulance vehicles, rural health centers) [62]. The study also performed a comparison regarding the performance of communication media such as GSM and satellite, which indicates that satellite communication performs well when handling a large file size [62].

### Conclusions

Despite the increasing number of professionals, fishermen, and merchant seafarers, maritime working conditions are characterized by an absence of access to health care facilities. This condition is further aggravated for seafarers who are working in the Arctic regions. Even if telemedicine has seen success onshore, there is limited success to it offshore. This is due to the absence of a good communication network, lack of trained personnel (paramedics), lack of maritime and offshore enhanced telemedicine equipment, bad weather conditions, and the distance and time required for SAR helicopters to reach a site, which reduces the possibility of MEDEVAC. Technology adoption from onshore to offshore settings might seem like a quick remedy for the case, but this remains a challenge for various reasons. The major challenge lies in the convergent and divergent nature of maritime and onshore telemedicine with respect to structure, practice, and policy. Therefore, it is necessary to identify these divergent and convergent points and to carefully review them before transferring technology and research experiences to offshore scenarios.

Despite these limitations, recently, a number of successes have been achieved in delivering telemedicine services in maritime settings, remote emergency responses, and Arctic and other extreme weather scenarios. These services include teleconsultation, teleradiology, telecardiology, tele-ENT, teledermatology, and tele-education, to mention a few. Most of these studies demonstrate the use of various means of communication including satellite, mobile, radio, and others. Moreover, all these studies have shown the use of various telemedicine modalities including video, still images, audio, and medical data. However, the use of telemedicine in relation to the search and rescue (SAR) services is not yet fully exploited. During this review, we did not see a paper that implemented telemedicine services for search and rescue (SAR) scenarios. Therefore, we foresee that once implemented and evaluated, these telemedicine services will serve as an underlying model for the successful deployment of the future telemedicine-assisted search and rescue (SAR) services. Even though the provision of telemedicine within the extreme weather (Arctic) and offshore scenarios calls for having good communication infrastructure and more Arctic-enhanced equipment, successful telemedicine services cannot be met with only these technologies. It needs an organization that is committed, motivated, and willing to invest in a project, while also being capable of mobilizing the human performance factors toward delivering the services.

### Practice Points

The following points are worth considering for a meaningful telemedicine solution in the context of maritime and extreme weather conditions as in the Arctic region:

First, maritime and onshore telemedicine can be convergent and divergent with respect to structural, practical, and policy differences. Therefore, it is necessary to identify these differences and carefully review them before transferring technology and research experiences to offshore scenarios.

Second, sometimes evacuation might become difficult in the Arctic region; therefore, it is necessary to consider telemedicine as the actual health care delivery services, rather than simply considering it as means of information exchange.

Third, it is necessary to do more Arctic-enhanced telemedicine research and also to assess and analyze the telemedicine solutions deployed in other regions such as Antarctica.

Fourth, it is necessary to have a systematic analysis of previous accidents in the Arctic region in order to provide a knowledge base with respect to emergency preparedness and response, focusing on the various phases and types of accidents.

Fifth, it is important to analyze the status quo of search and rescue operation in the Arctic region, so as to identify the capability gaps and to take the necessary measures.

Sixth, as applying the current open water Escape, Evacuation, and Rescue technology might have an unacceptably high failure rate, it is necessary to deploy more Arctic-enhanced EER technology.

Seventh, in most of the literature, we have observed the lack of a common international standard or protocol for information sharing, that is, DICOM (Digital Imaging and Communication in Medicine). However, we have noticed some articles [50,62] that were based on the development of “Vital” and “DICOM” standards. Therefore, it is necessary to adopt such kinds of standard for an interoperable information communication.

Finally, it is necessary to consider the capability approach in the broad concept of integrated operation for offshore telemedicine services.

## Appendix

Multimedia Appendix 1 Literature included in the study, sorted from the latest to the oldest.

## Abbreviations

CDMA: code division multiple access
DICOM: Digital Imaging and Communication in Medicine
EER: Escape, Evacuation, and Rescue
ENPs: emergency nurse practitioners
GSM: Global System for Mobile Communication
GPRS: General Packet Radio Service
ISDN: Integrated Services Digital Network
MEDEVAC: medical evacuation
RHC: remote health care
SAR: search and rescue
VPN: Virtual Private Network
VSAT: very small aperture terminal
