# Supplementary material for: Telemedicine Services for the Arctic: A Systematic Review
Source: JMIR Med Inform. 2017 Jun 28;5(2):e16. doi: 10.2196/medinform.6323 (PMC5508113; doi:10.2196/medinform.6323)
Supplement: Multimedia Appendix 1 [file medinform_v5i2e16_app1.pdf]

Table 1. Literature included in the study, sorted from the latest to the oldest.

Reference	System/Company/ Project	Type of circumstance	Communication link	Telemedicine modalities	Telemedicine services
[52]	A real-time emergency telemedicine system	[a] Emergency or remote	Red hybrid radio, modem Internet (ADSL, fiber optics, mobile phones)	Transmission of biomedical data	Telemonitoring, tele-ambulance
[46]	Remote Health Care Strategy (RHC) (Shell's Medical Emergency Response)	Arctic or remote	Satellite	Still images, videoconferencing	Teleradiology, real-time video consultation, tele-ENT
[63]	Upstream Nigeria Telemedicine Pilot	Emergency or remote	Mobile	Vendor-NuPhysicia (video, still images)	Real-time teleconsultation (ENT exam, discuss lab result, discuss prescribed medications)
[12]	The future of Telemedicine in O&G, 2013	Emergency or remote and offshore	[b]-----	Dedicated software	Real-time data sharing and decision-making
[64]	Tele medical assistance services (TMAS)	Maritime or offshore	Satellite	e-mail and still images	Teledermatology
[45]	Statoil	Emergency or remote and offshore	Private LAN or broadband	Video conferencing	Real-time teleconsultation and telesonography
[33]	TCP (Saipem Telecardiology Program)	Emergency or remote and offshore	Dial-Up	Telephone and e-mail (written recommendations as PDF)	Telecardiology
[41]	Kashagan Project	Emergency or remote and offshore	Satellite	Videoconferencing (Skype), e-mail, and still images	Real-time teleconsultation, telecardiology
[31]	Centro Internazionale Radio Medico (CIRM)	Maritime or offshore	Satellite	Radio and telephone, e-mail	Teleconsultation and Telemedical Maritime Assistance Service (TMAS)
[65]	Eni Oil and Gas company	Remote	GSM, PSTN, ISDN, LAN, satellite	E-mail, telephone, still images	Telecardiology, asynchronous teleconsultation, real-time data sharing
[66]	Emergency medical services (EMSs)	Emergency	Local network (Peeq-Box) and a server (Asterisk, Digium Inc)	Continuous audio, vital data, and video transmission	Tele-EMS
[39]	French Tele medical Assistance Service (TMAS)	Emergency or maritime or offshore	-----	Telephone, still images, videoconference with Skype	Teleconsultation, telecardiology, real-time video consultation, telephone advice

a "**or ,**" is used when an article considers more than one circumstances, communication link, telemedicine modalities and telemedicine services.

b "-----" Depicts missing data or description in the article.

([48 ,49])	Japan Syowa station	Antarctica or , emergency or remote	Radiotelegraph, radio, satellite radio-telephone and satellite radio facsimile, mobile	E-mail, still and moving pictures (video)	Real-time teleconsultation, teleradiology, teledermatology, tele-orthopedics, tele-ophthalmology, internal medicine, tele-urology, and teledentistry
[57]	Emergency medical service (EMS)	Emergency or remote	Peeq-Box, P3 communication, mobile networks	E-mail, video, audio, Still images, fax, vital data	Teleconsultation, tele-EMS
[56]	Sonography for Trauma examination	Emergency or remote	Radio	Video and 2-way radio	Tele-ultrasound, telesonography
[28]	Tempus IC Telemedicine	Emergency or remote and offshore	Satellite, Internet, or mobile	Voice, images, data, and video	-----
	InPlace Medical	Emergency or remote and offshore	-----	Videoconferencing, still image, and audio	Teledermatology, tele-ENT
[44]	Amazon Virtual Medical Team (AVMT)	Remote or maritime or offshore	Satellite, mobile	Videoconferencing (Skype), satellite phone, telephone	Telepresence, real-time teleconsultations
[38]	Scorpion Offshore	Emergency or maritime or offshore	Rig bandwidth	Video conferencing	Real-time specialty heart visit and urgent care visit (examine, manage, and treat), tele-ENT, teledermatology
[67]	Aberdeen Royal Infirmary	Maritime or offshore	NHS network	E-mail, still images, and telephone	Electrocardiogram (ECG) interpretation and advice (TMAS)
[40]	Alwyn North oil platform operated by Total	Maritime or offshore	Satellite	Videoconferencing	Real-time teleconsultation and telesonography
[55]	Minor injuries telemedicine service (Aberdeen ED)	Emergency	-----	Videoconferencing	Teleconsultation
[37]	Minimum medical emergency data-set (MMEDS)	Remote	Virtual private network (VPN), mobiles	Data,<br>SMS messages	Real-time data sharing and decision-making
[62]	“Multi-purpose” and “all-weather” telemedicine system	Emergency or remote or offshore	GSM, GPRS, Satellite and Plain Old Telephone System (POTS)	Still images	Bidirectional telepointing capability, images, and real-time one ECG lead waveform transmission
[60]	(CDMA)-based emergency telemedicine system (CETS)	Emergency or remote	CDMA-based networks (Mobile)	Bio signal transmission	Telemonitoring, tele-ambulance
[61]	Mobile telemedicine unit (MTU)	Emergency or Arctic	Mobile	Still images	Teleradiology, tele-ECG, tele-electroencephalography, asynchronous teleconsultation
[47]	British Antarctic Survey Medical Unit (BASMU)	Antarctica or emergency or remote	Radio telephone, satellite	Still images, e-mail, fax, telephone	Teleradiology, teledermatology, tele-interpretation, telespirometry, real-time consultation, real-time data sharing and decision-making, thrombolysis, real-time Telemonitoring, tele-education (training)

[51]	Arctic health information and research	Arctic	Satellite, mobile, radio	Video conferencing	Tele-education (Training)
[50]	Italian Antarctic Base at Terra Nova Bay	Antarctica or emergency or remote	INMARSAT (Satellites), mobile phones, VPN	Medical data, still images, e-mail, fax, video	Videoconference, teleconsultation, teleradiology, on-line and off-line teleconsultation (ophthalmic, radiology, and orthopedic)
[32]	Aberdeen Royal Infirmary’s accident and emergency department	Emergency or remote or offshore	Radio	Radio and telephone	Radio medical advice
[59]	Moorfields Eye Hospital	Accident and emergency	----- (Not specified (384 kbits per second))	Videoconferencing, telephone	Real-time teleconsultations, tele-ophthalmology
[58]	Aberdeen accident and emergency department	Accident and emergency or remote	ISDN at 384 kbits per second	Videoconferencing, still images	Real-time teleconsultations, teleradiology
[68]	Military treatment facilities (MTFs)	Maritime or offshore	NMCSD (“hub-and-spoke”)	Videoconferencing	ENT teleconsultations
[34]	MERMAID	Remote or maritime, offshore or emergency	ISDN, digital land lines, cellular or wireless, satellite and broadband	Videoconferencing	Tele-education (Training)
[4]	Alaska Telemedicine Project	Arctic or remote	Single-side band radio, satellite radio, and television	Videoconferencing, medical messaging and internet, e-mail, still images	Clinical decision-support, teleradiology, tele-ENT and dermatology
	Turku University hospital	Arctic or remote	ISDN	Still images	Teleradiology
	Greenland	Arctic	-----	Video, still images	Videoconsultations, teleradiology
	Arkhangelsk Region (PC-based system)	Arctic or remote	-----	Still images, audio-telephone	Teleconsultations, tele-education (Training)
[43]	Mount Logan and Mount McKinley Telemedicine Projects	Arctic or remote	Mobile satellite communication	Physiological data	Telemonitoring
	Baffin Telehealth Project	Arctic or remote	Satellite	Video, still images	Real-time teleconsultation, videoconferencing, digital imaging, and various medical diagnostics
[35]	MERMAID	Remote or maritime, offshore or emergency	ISDN, digital land lines, cellular/wireless, satellite, and broadband	Videoconferencing, audio, multimedia, flat files, and still images	Telepresence, real-time teleconsultations
[26]	MERMAID	Remote or maritime, offshore or emergency	ISDN, digital land lines, cellular/wireless, satellite, and broadband	Videoconferencing, audio, multimedia, flat files, and still images	Telepresence, real-time teleconsultations

Reference

4. Hild M. Arctic Telemedicine Project Final Report. Presented to the Sustainable Development Working Group of the Arctic Council. Institute for Circumpolar Health Studies, University of Alaska Anchorage: Anchorage, Alaska 2000:97. URL: http://hdl.handle.net/11374/27.
12. Thorvik K, Nystad A, Fernandes A, Reegard K, Simensen J, Rindahl G, Silva E, Bergsland T, Klingsheim O, Evjemo T. The future of telemedicine in O&G. SPE Intelligent Energy Conference & Exhibition; 1-3 April 2014; Utrecht, The Netherlands: Society of Petroleum Engineers; 2014. doi: 10.2118/167841-ms.
26. Anogianakis G, Maglavera S, Pomportsis A. Relief for maritime medical emergencies through telematics. IEEE Transactions on Information Technology in Biomedicine. 1998;2(4):254-60. doi: 10.1109/4233.737580.
28. Anscombe DL. Healthcare delivery for oil rig workers: telemedicine plays a vital role. Telemedicine journal and e-health: the official journal of the American Telemedicine Association. 2010;16(6):659-63. doi: 10.1089/tmj.2010.9957. PubMed PMID: 20690836.
31. Amenta F, Capone L, Sibilio F. Telemedical Assistance of Patients on Board Ships Activity of Centro Internazionale Radio Medico (CIRM), the Italian Telemedical Maritime Assistance Service (TMAS). 2013. URL: http://www.cirm.it/documenti/cirm_th3-1.pdf [accessed 2017-06-05] [WebCite Cache ID 6qzOLTH3p]
32. Aujla K, Nag R, Ferguson J, Howell M, Cahill C. Rationalizing radio medical advice for maritime emergencies. Journal of Telemedicine and Telecare. 2003;9(1_suppl):12-4. doi: 10.1258/135763303322196178. PubMed PMID: 12952706.
33. Panait DC, Mika F. Tele-Cardiology in Remote O&G Premises. European HSE Conference and Exhibition; 2013/4/16/; London, United Kingdom. SPE: Society of Petroleum Engineers; 2013. doi: 10.2118/164984-ms.
34. Anogianakis G, Maglavera S. Utilising multimedia for training merchant mariners as paramedics. User Acceptance of Health Telematics Applications. 2000; 72:66-72. doi: 10.3233/978-1-60750-916-5-66. PubMed PMID: WOS:000086539700010.
35. Anogianakis G, Maglavera S, Pomportsis A, Bountzioukas S, Beltrame F, Orsi G. Medical emergency aid through telematics: design, implementation guidelines and analysis of user requirements for the MERMAID project. International Journal of Medical Informatics. 1998;52(1):93-103. doi: 10.1016/s1386-5056(98)00128-2.
37. Anogeianaki A, Papaliagkas V, Guibas G, Anogianakis G. Telemedicine services across the Greek-Bulgarian border. Journal of Telemedicine and Telecare. 2007;13(3_suppl):1-3. doi: 10.1258/135763307783247248.
38. Boultinghouse OW, Fitts Jr TG. Telemedicine technologies enhance offshore healthcare, reduce illness-related departures. Drilling contractor. 2009. URL: http://www.drillingcontractor.org/telemedicine-technologies-enhance-offshore-healthcare-reduce-illness-related-departures-1853 [accessed 2017-05-26] [WebCite Cache ID 6qkkKUSKg]
39. Dehours E, Vallé B, Bounes V, Girardi C, Tabarly J, Concina F, Pujos M, Ducassé J-L. User satisfaction with maritime telemedicine. Journal of Telemedicine and Telecare. 2012;18(4):189-92. doi: 10.1258/jtt.2012.110910. PubMed PMID: 22604271.
40. Mair F, Fraser S, Ferguson J, Webster K. Telemedicine via satellite to support offshore oil platforms. Journal of Telemedicine and Telecare. 2008;14(3):129-31. doi: 10.1258/jtt.2008.003008. PubMed PMID: 18430278.
41. Kevlishvili G, Mika F, De Sanctis S. Trial Teleconsultation Sessions On Castoro 12 In North Caspian Sea. SPE European Health, Safety and Environmental Conference in Oil and Gas Exploration and Production; 2011/1/1/; Vienna, Austria. SPE: Society of Petroleum Engineers; 2011. doi: 10.2118/140501-ms.
43. Otto CA. Telemedicine in the Canadian high Arctic and other remote environments. In: Engineering in Medicine and Biology 21st Annual Conference and the 1999 Annual Fall Meetring of the Biomedical Engineering Society BMES/EMBS Conference.: IEEE; 1999 Presented at: Proceedings of the First Joint BMES/EMBS Conference. 1999 IEEE Engineering in Medicine and Biology 21st Annual Conference and the 1999 Annual Fall Meeting of the Biomedical Engineering Society (Cat. N; October 1999; Atlanta, GA URL: http://ieeexplore.ieee.org/document/803864/ [doi: 10.1109/IEMBS.1999.803864]
44. Latifi R, Stanonik Mde L, Merrell RC, Weinstein RS. Telemedicine in extreme conditions: supporting the Martin Strel Amazon Swim Expedition. Telemedicine journal and e-health: the official journal of the

American Telemedicine Association. 2009;15(1):93-100. doi: 10.1089/tmj.2008.0057. PubMed PMID: 19199853.
45. Todnem K, Evensen A, Oveland N. The implementation of telemedicine as an integrated part of the health service on the Statoil operated installations on the Norwegian continental shelf (NCS). International Conference on Health, Safety and Environment in Oil and Gas Exploration and Production; 2012/1/1/; Perth, Australia. SPE: Society of Petroleum Engineers; 2012. doi: 10.2118/157562-ms.
46. Berg J, Toner S, Stilz R, Klein S, Williams H, Pearson J, Fraser A, Norman N. Remote Health Care: A Game Changer for the Arctic. OTC Arctic Technology Conference; 2015 2015/3/23/; Copenhagen, Denmark. OTC: Offshore Technology Conference. doi: 10.4043/25549-ms.
47. Grant IC. Telemedicine in the British Antarctic survey. International Journal of Circumpolar Health. 2004;63(4):356-64. PMID:15709312.
48. Ohno G. Practical Results of Telemedicine System Between Antarctic Station and Japan. In: Graschew G, Rakowsky S. Telemedicine Techniques and Applications. Rijeka: InTech; 2011. p. Ch. 21.
49. Ohno G, Watanabe K, Okada Y, Higuchi K. Practical experience of telehealth between an Antarctic station and Japan. Journal of Telemedicine and Telecare. 2012;18(8):473-5. doi: 10.1258/jtt.2012.GTH111. PubMed PMID: 23209273.
50. Pillon S, Todini AR. eHealth in Antarctica: a model ready to be transferred to every-day life. International Journal of Circumpolar Health. 2004;63(4):436-42. PMID: 15709319.
51. Hild C. Arctic telehealth: North to the future. International Journal of Circumpolar Health. 2004;63(sup2):63-70. PMID:15736624.
52. Castellano NN, Gazquez JA, García Salvador RM, Gracia-Escudero A, Fernandez-Ros M, Manzano-Agugliaro F. Design of a real-time emergency telemedicine system for remote medical diagnosis. Biosystems Engineering. 2015; 138:23-32. doi: 10.1016/j.biosystemseng.2015.03.017.
55. Miller DR, Alam K, Fraser S, Ferguson J. The delivery of a minor injuries telemedicine service by Emergency Nurse Practitioners. Journal of Telemedicine and Telecare. 2008;14(3):143-4. doi: 10.1258/jtt.2008.003013. PubMed PMID: 18430283.
56. Boniface KS, Shokoohi H, Smith ER, Scantlebury K. Tele-ultrasound and paramedics: real-time remote physician guidance of the Focused Assessment with Sonography for Trauma examination. The American Journal of Emergency Medicine. 2011;29(5):477-81. doi: 10.1016/j.ajem.2009.12.001. PubMed PMID: 20825815.
57. Bergrath S, Reich A, Rossaint R, Rörtgen D, Gerber J, Fischermann H, Beckers SK, Brokmann JC, Schulz JB, Leber C, Fitzner C, Skorning M. Feasibility of Prehospital Teleconsultation in Acute Stroke – A Pilot Study in Clinical Routine. PLoS ONE. 2012;7(5): e36796. doi: 10.1371/journal.pone.0036796. PubMed PMID: 22629331; PubMed Central PMCID: PMCPMC3356340.
58. Brebner EM, Brebner JA, Ruddick-Bracken H, Wootton R, Ferguson J. Evaluation of a Pilot Telemedicine Network for Accident and Emergency Work. Journal of Telemedicine and Telecare. 2002;8(2_suppl):5-6. doi: 10.1258/135763302320301803. PubMed PMID: 12217114.
59. Bowman RJ, Kennedy C, Kirwan JF, Sze P, Murdoch IE. Reliability of telemedicine for diagnosing and managing eye problems in accident and emergency departments. Eye. 2003;17(6):743-6. doi: 10.1038/sj.eye.6700489. PubMed PMID: 12928688.
60. Kang J, Chun H, Shin IH, Shin SD, Suh GJ, Kim HC. Preliminary evaluation of the use of a CDMA-based emergency telemedicine system. Journal of Telemedicine and Telecare. 2006;12(8):422-7. doi: 10.1258/135763306779378744. PubMed PMID: 17227609.
61. Uldal SB, Amerkhanov J, Manankova Bye S, Mokeev A, Norum J. A mobile telemedicine unit for emergency and screening purposes: experience from north-west Russia. Journal of Telemedicine and Telecare. 2004;10(1):11-5. doi: 10.1258/135763304322764121. PubMed PMID: 15006209.
62. Kyriacou E, Pavlopoulos S, Koutsouris D. An Emergency Telemedicine System Based on Wireless Communication Technology: A Case Study. In: Istepanian RSH, Laxminarayan S, Pattichis CS, editors. M-Health: Emerging Mobile Health Systems. Boston, MA: Springer US; 2006. p. 401-16.
63. Dim S, Aliyu B. Upstream Nigeria Telemedicine Pilot Program. SPE International Conference on Health, Safety, and Environment; 2014/3/17/; Long Beach, California, USA. SPE: Society of Petroleum Engineers; 2014. doi: 10.2118/168565-ms.
64. Dahl E. Briefing notes on maritime teledermatology. Int Marit Health. 2014;65(2):61-4. doi: 10.5603/IMH.2014.0014. PubMed PMID: 25231327.

65. Mortara S, Uberti F, Gomez G, editors. Telemedicine in O&G Industry: Our Experience (Russian). SPE Russian Oil and Gas Technical Conference and Exhibition; 2008 2008/1/1/; Moscow, Russia. SPE: Society of Petroleum Engineers. doi: 10.2118/115505-ru.
66. Rörtgen D, Bergrath S, Rossaint R, Beckers SK, Fischermann H, Na I-S, Peters D, Fitzner C, Skorning M. Comparison of physician staffed emergency teams with paramedic teams assisted by telemedicine – a randomized, controlled simulation study. Resuscitation. 2013;84(1):85-92. doi: 10.1016/j.resuscitation.2012.06.012. PubMed PMID: 22750663.
67. Webster K, Fraser S, Mair F, Ferguson J. A low-cost decision support network for electrocardiograph transmission from oil rigs in the North Sea. Journal of Telemedicine and Telecare. 2008;14(3):162-4. doi: 10.1258/jtt.2008.003021. PubMed PMID: 18430291.
68. Melcer T, Hunsaker D, Crann B, Caola L, Deniston W. A prospective evaluation of ENT telemedicine in remote military populations seeking specialty care. Telemedicine journal and e-health: the official journal of the American Telemedicine Association. 2002;8(3):301-11. doi: 10.1089/15305620260353199. PubMed PMID: 12419024.
